# Optimization of Duplex Stability and Terminal Asymmetry for shRNA Design

**DOI:** 10.1371/journal.pone.0010180

**Published:** 2010-04-20

**Authors:** Olga V. Matveeva, Yibin Kang, Alexey N. Spiridonov, Pål Sætrom, Vladimir A. Nemtsov, Aleksey Y. Ogurtsov, Yury D. Nechipurenko, Svetlana A. Shabalina

**Affiliations:** 1 Department of Human Genetics, University of Utah, Salt Lake City, Utah, United States of America; 2 Department of Molecular Biology, Princeton University, Princeton, New Jersey, United States of America; 3 Department of Applied Mathematics, Massachusetts Institute of Technology, Cambridge, Massachusetts, United States of America; 4 Department of Computer and Information Science, Norwegian University of Science and Technology, Trondheim, Norway; 5 Department of Cancer Research and Molecular Medicine, Norwegian University of Science and Technology, Trondheim, Norway; 6 MGGT, Russian Academy of Sciences, Moscow, Russia; 7 Engelhardt Institute of Molecular Biology, Russian Academy of Sciences, Moscow, Russia; 8 National Center for Biotechnology Information, National Library of Medicine, National Institutes of Health, Bethesda, Maryland, United States of America; National University of Singapore, Singapore

## Abstract

Prediction of efficient oligonucleotides for RNA interference presents a serious challenge, especially for the development of genome-wide RNAi libraries which encounter difficulties and limitations due to ambiguities in the results and the requirement for significant computational resources. Here we present a fast and practical algorithm for shRNA design based on the thermodynamic parameters. In order to identify shRNA and siRNA features universally associated with high silencing efficiency, we analyzed structure-activity relationships in thousands of individual RNAi experiments from publicly available databases (ftp://ftp.ncbi.nlm.nih.gov/pub/shabalin/siRNA/si_shRNA_selector/). Using this statistical analysis, we found free energy ranges for the terminal duplex asymmetry and for fully paired duplex stability, such that shRNAs or siRNAs falling in both ranges have a high probability of being efficient. When combined, these two parameters yield a ∼72% success rate on shRNAs from the siRecords database, with the target RNA levels reduced to below 20% of the control. Two other parameters correlate well with silencing efficiency: the stability of target RNA and the antisense strand secondary structure. Both parameters also correlate with the short RNA duplex stability; as a consequence, adding these parameters to our prediction scheme did not substantially improve classification accuracy. To test the validity of our predictions, we designed 83 shRNAs with optimal terminal asymmetry, and experimentally verified that small shifts in duplex stability strongly affected silencing efficiency. We showed that shRNAs with short fully paired stems could be successfully selected by optimizing only two parameters: terminal duplex asymmetry and duplex stability of the hypothetical cleavage product, which also relates to the specificity of mRNA target recognition. Our approach performs at the level of the best currently utilized algorithms that take into account prediction of the secondary structure of the target and antisense RNAs, but at significantly lower computational costs. Based on this study, we created the si-shRNA Selector program that predicts both highly efficient shRNAs and functional siRNAs (ftp://ftp.ncbi.nlm.nih.gov/pub/shabalin/siRNA/si_shRNA_selector/).

## Introduction

siRNAs are short duplexes consisting of the antisense (cleavage guidance) and the sense (passenger) strand where antisense strands are complementary to their RNA targets and specifically silence gene expression. They can be designed by researchers to silence particular genes of interest but only some successfully do so. The accumulation of published gene silencing experimental data makes the task of designing highly efficient and specific siRNAs very appealing and a number of sophisticated models for predicting siRNA efficiency have been created [1]–[8] and compared with each other [9]–[11].

siRNA-mediated silencing of mammalian genes uses synthetic oligonucleotides transfected into cells. An alternative approach employs expression of short hairpin RNAs (shRNAs) in cells after delivery of expression plasmids or viral vectors [12]. shRNAs are artificial analogs of endogenous miRNAs, the vast class of small non-coding RNA molecules that regulate stability and translation of their target mRNAs. Precursors of miRNAs (pre-miRNAs) are stable hairpins which are encoded in plant and animal genomes (see reviews [13], [14]). The relative duplex instability at the 5′ end of the RNA antisense strand facilitates its preferential incorporation into the RNA Inducible Silencing Complex (RISC) [15], [16]. The selective assembly of the antisense strand into RISC probably reflects the relative ease of unwinding from one end of the antisense-sense duplex. The thermodynamic properties of the miRNA-like and siRNA-like duplexes, such as terminal end stability measured through Gibbs free energy evaluation, determine the asymmetrical RISC assembly and, therefore, the efficiency of target gene silencing. Since processing of artificial siRNAs and shRNAs in cells utilizes the main components of cellular RNAi machinery, design of these molecules should allow provision for successful interaction with RISC and mRNA targets.

Many popular designs of miRNA-like shRNAs are based on Drosha and Dicer-mediated cleavage and are used for the loss-of-function assays [17], [18]. The processing of shRNAs with long stems (22 nucleotides or more) depends strongly on Dicer activity. It was suggested, however, that the processing of shRNAs from shorter (19 nucleotide) stems, is not Dicer-dependent [19]. Perhaps single strand RNases (for example, representatives of RNase A gene super family [20]) are involved in the processing of shRNAs with short stems. Thus, the ability of shRNAs to silence genes might depend on the susceptibility of their loop sequences to cleavage by single strand RNases. This hypothesis is strengthened by the observation that the main distinguishable features between very efficient and completely inefficient molecules in gene silencing experiments with short stem (∼19 nucleotides) shRNAs were loop lengths [12] and loop nucleotide content (P.M. Chumakov personal communication).

The approach to gene silencing based on synthesized siRNAs is fast and simple. The shRNA based approach is more laborious and time consuming, but it is becoming increasingly popular. Compared to chemically synthesized siRNAs, the shRNA approach offers advantages in silencing longevity and lower costs for genome-wide studies. Also, gene therapy is a particularly promising application for shRNAs. It is believed that transcription of shRNA delivers lower intra-cellular concentrations of siRNA-like products, compared to synthetic siRNA oligonucleotides transfected into cells. Lower intra-cellular concentrations achieved through the natural process of transcription for extended periods of time can yield more specific silencing effects.

Current predictive models of siRNA behavior are frequently used for shRNA design, however, many of them fail to discriminate between efficient and inefficient shRNA [21]. To define and optimize factors universally related to siRNA and shRNA efficiency, we analyzed four independent siRNA databases and one database of shRNA constructs. We found that optimization of thermodynamic stability and the terminal asymmetry in stability of the fully paired duplex for small RNA antisense strand allows the selection of both siRNAs and shRNAs with high efficiency.

To verify the predictive power of our approach, we designed 83 shRNAs and performed experiments which demonstrated that efficient shRNAs with short fully paired stems could be successfully selected by optimization of the terminal and general duplex stability of the hypothetical cleavage products.

## Materials and Methods

### Experimental databases

The following shRNA databases were used in our analysis: the Netherlands Cancer Center shRNA database (277 shRNAs); shRNA subset from siRecords database (642 shRNAs); Princeton University shRNA database (83 shRNAs).

The following siRNA databases were used in our analysis**: Novartis siRNA database** [2431 experimental data points [6]]; **Sloan Kettering siRNA database** [601 experimental data points[22]]; **University of Tokyo siRNA database** [702 experimental data points[23]]; **NCBI database** [652 experimental data points [3]].

In Novartis, Sloan Kettering and University of Tokyo siRNA databases, gene down regulation was achieved through siRNA oligonucleotide transfection and the remaining protein levels were evaluated through fluorescence measurement of reporter gene output. In NCBI database gene down regulation was achieved through siRNA oligonucleotide transfection and the remaining RNA or protein levels were evaluated by different assays.

**University of Minnesota siRecords database** (http://siRecords.biolead.org) includes several thousands of mammalian RNAi experimental data points compiled from different sources, but only 642 shRNA experiments were chosen for the analysis in this study. We choose data that represent vector based shRNA intracellular delivery with target mRNA sequence of 19 nucleotides in length. From this data we further choose data for which gene down-regulation was evaluated through measurement of the relevant mRNA level. For data analysis, we substituted silencing efficiency category titles with percentage of remaining target RNA values. The “very high” category was substituted with 5% of the remaining target RNA, “high”- with 20%, “medium” - with 40% and “low” - with 100%.

**Princeton University shRNA database** includes 83 experiments performed for this study. shRNAs were expressed in MDA-MB-231 breast cancer cell line using retroviral vector (produced from constructs of plasmid “pSuper.retro.puro” (OligoEngine.Inc,WA 98103)) and the gene down-regulation was evaluated through Northern blot or qRT-PCR. 79 constructs were designed to form 20 nucleotide duplexes of potential siRNA–like cleavage products, and 4 constructs were designed to form 19 nucleotide duplexes. All shRNA constructs were designed to target variable cellular genes using viral transduction. All constructs were designed with asymmetry in terminal local duplex stability of potential siRNA like cleavage product being equal or above of 2 kcal/mol. The ΔΔG value was calculated by subtraction ΔG of 3′ from ΔG of 5′ termini of antisense strand of RNA duplex. Sometimes “U-G” wobble pair was used at the end of the duplex with “U” located in the sense strand and “G” in the antisense strand to achieve correct asymmetry. The nine nucleotide loop sequence in the hairpin structure transcript was 5′-UUCAAGAA-3′.

### Computer analysis of parameters and selection thresholds

Thermodynamic thresholds suggested in this study for efficient siRNA or shRNA design were chosen by a trial and error approach considering trade-offs between specificity and sensitivity of prediction.

The calculations related to the local and terminal duplex stability were performed with thermodynamic parameters published earlier [24]. Terminal siRNA duplex asymmetry (ΔΔG) was calculated for the two terminal nucleotide base pairs of each siRNA duplex by subtracting the value for the 5′ sense strand from that of the 5′ antisense strand. “RNA structure” package [25] (version 4.6) was used for calculations of ΔG values related to stabilities of siRNA duplexes, antisense strand hairpins and local target RNA secondary structures. RNA folding stability for target mRNAs and small RNA antisense strands was also estimated by Afold program [26], [27]. Calculations related to siRNA duplex stability, antisense strand hairpins and local secondary structures of RNA targets, produced slightly different ΔG values for individual siRNAs depending on whether version 3.7 or 4.6 of the package RNA structure was used. Nevertheless, the correlation coefficients or categorization results were almost identical regardless of the version used. Categorization according to RNA secondary structure stability was performed for siRNA and shRNA data subsets with optimal terminal duplex asymmetry (ΔΔG ≥2 kcal/mol). ΔG cost of RNA unfolding calculated as the difference of free energy between optimally folded and completely single stranded local mRNA target was evaluated by two different programs [25]). Categorization by these two different programs demonstrated similar results (data shown for folding algorithm by Mathews et al. [25]).

Excel 2007 (Microsoft, Inc.) was used for correlation analysis and graphical data presentation. Statistical analysis was performed using Excel Macros “Average” created for this study which allows categorization of experimental data-points from a database according to independent variable intervals defined by user and calculation of averaged dependant variable for each interval. We used ΔG values as independent variables and remaining level of si-shRNA targeted mRNA or protein in cells as dependant variable. ROC analysis was performed using “MedCalc” (http://www.medcalc.be/contact.php) and “Anylise-it” (http://www.analyse-it.com/).

## Results

### Optimization of duplex terminal asymmetry

For successful gene silencing experiments, antisense strand RISC loading is desirable while loading of the sense strand can direct non-specific gene silencing. The two strands of siRNA duplexes can compete with each other for loading into RISC [15]–[16]. Optimization of terminal asymmetry in local duplex stability can help to achieve higher efficiency and strand selectivity of gene silencing.

It is a challenging task to define quantitative relationship between stabilities of 5′ pairing of siRNA strands and their RISC entry reaction rates. Based on experimental estimation of free energy of transition complex formation, we created a model that describes siRNA strands entries into the RISC complex (Figure S1). According to this model, the formation energy of the transition complex and the loading rate depend exponentially on the thermodynamic stability of terminal 5′ nucleotide base pairs in a siRNA duplex as measured using Gibbs free energy value. Consequently, in the case of siRNA, reaction rate should depend exponentially on stability of the terminal 5′ nucleotide base pairs. As far as this stability is evaluated using Gibbs free energy value, ΔΔG (the subtraction value of ΔG related to the 5′ ends of the antisense and sense strands) determines the proportion of RISC entry rates for the antisense and sense strands. This proportion should also depend exponentially on ΔΔG. Eyring's transition state theory provides physical justification for using ΔG difference instead of other comparisons between terminal ΔG values (e.g. their ratio) in estimating the terminal siRNA duplex asymmetry (Figure S1).

Graphical presentation of the relationship between ΔΔG and reaction rate difference is shown in Figure S1. This theoretical relationship is in good agreement with experimental estimations inferred from rates of target cleavage by Schwarz et al., 2003 [16]. It is seen from the Figure that ΔΔG values equal to or higher than 2 kcal/mol correspond to ∼20 or more times higher difference between RISC loading rates for the antisense versus sense strand. Thus, 2 kcal/mol could be considered as a threshold for substantial domination of antisense strand entry into the RISC complex.

Substitutions that affect silencing efficiency can be located within the first four nucleotides of the 5′ end [16], however, two terminal nucleotides have a strongest effect on the silencing efficiency [3], [10], [28]. Our computer analysis of several experimental databases also confirmed that the first two nucleotides play a major role in defining shRNA efficiency as well as siRNA efficiency. For example, ROC analysis of four independent siRNA databases used for this study further confirmed this observation (Table 1). Thus, we defined and calculated terminal duplex asymmetry as Gibbs free energy difference for two terminal nucleotides in this study.

**Table 1:** Optimization of terminal duplex asymmetry.

Database	Area under ROC curve for ΔΔG (2 nt)	Significance	Area under ROC curve for ΔΔG (4 nt)	Significance
Novartis	0.62	<0.001	0.61	<0.001
Sloan Kettering	0.7	<0.001	0.67	<0.001
University of Tokyo	0.62	<0.001	0.61	<0.001
NCBI	0.67	<0.001	0.65	<0.001
siRECORDS from University of Minnesota	0.55	0.0098	0.52	not significant
Netherland Cancer Institute	0.6	0.082	0.5	not significant

ROC analysis of shRNA and siRNA databases considering four and two terminal base pairs.

**Figure 1:**
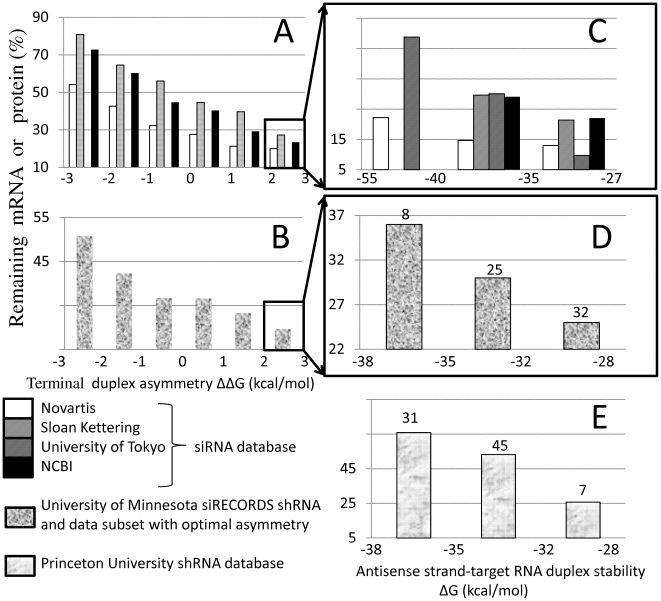
Relationships between silencing efficiency and thermodynamic factors. Silencing experiments were categorized according to asymmetry of the terminal duplex stability measured as ΔΔG or stability of RNA duplex measured as ΔG. The average amount of target mRNA or remaining relevant protein was calculated for each category. The number of representatives in each data category is indicated above each column for shRNA data subsets. Detailed information for these siRNA and shRNAs datasets is presented in Table S1. a. siRNAs from four different databases categorized according to asymmetry of the terminal local duplex stability measured as ΔΔG value. Correlation coefficients for relationships between remaining levels of mRNA or protein and terminal duplex asymmetry in stability (ΔΔG) are *R* = 0.47, *p* = 1.1*10^−136^ for Novartis, *R* = 0.47, *p* = 2.8*10^−34^ for Sloan Kettering, *R* = 0.29, *p* = 2.9*10^−15^ for University of Tokyo, *R* = 0.39, *p* = 4.7*10^−25^ for NCBI database. b. shRNA experiments (642 sequences) from siRECORDS University of Minnesota database categorized according to asymmetry of the terminal local duplex stability measured as ΔΔG value (Correlation coefficient for relationship between remaining level of RNA and terminal duplex asymmetry in stability (ΔΔG) is *R* = 0.13, *p* = 10^−3^). c. siRNAs with optimal terminal duplex asymmetry (ΔΔG ≥2 kcal/mol) from four different databases categorized according to the antisense strand-target duplex stability measured as ΔG value (correlation coefficients and significance values for relationships between remaining level of mRNA or protein levels and ΔG values are indicated in Table S2). d. shRNAs with the optimal terminal duplex asymmetry (ΔΔG ≥2 kcal/mol) from siRECORDS University of Minnesota database (642 sequences) categorized according to the antisense strand-target duplex stability measured as ΔG value (correlation coefficient and significance value for relationship between remaining mRNA level and ΔG value are *R* = 0.35, *p* = 0.9*10^−3^). e. shRNAs from Princeton University experiments categorized according to the antisense strand-target duplex stability measured as ΔG value. All shRNA were designed to produce potential siRNA–like cleavage product with the optimal terminal asymmetry (ΔΔG ≥2 kcal/mol) (correlation coefficient and significance value for relationship between remaining mRNA level and ΔG value are *R* = 0.3, *p* = 0.007).

Data categorization according to this factor for siRNA and shRNA experiments is shown in Figure 1. Chart A in Figure 1 demonstrates that for all four independent siRNA experimental databases, the lowest level of remaining target RNA or protein is achieved in the category with ΔΔG values equal to, or higher than, 2 kcal/mol. The same effect was observed for the shRNA experimental database (Figure 1B). For silencing experiments, we suggest designing molecules with the terminal asymmetry in this range. However, this limitation is not necessary if siRNA duplexes contain 5′-modified passenger strands that block 5′-phosphorylation and, hence, RISC loading of the passenger strand.

### Optimization of duplex stability of fully paired siRNA antisense strand

How can we use duplex stability of fully paired siRNA or shRNA antisense strands (further, duplex stability) to predict silencing efficiency? Data categorization according to the duplex stability evaluated through the calculation of Gibbs free energy (ΔG) was performed for data subsets with terminal asymmetry of siRNA or potential siRNA–like cleavage product of 2 kcal/mol and higher. We found in the subset that includes data from four independent siRNA experimental databases the lowest level of remaining target RNA or protein is achieved in the category with ΔG values ranging from −35 to −27 kcal/mol (Figure 1C). We observed a similar effect for the shRNA data subset with optimal terminal duplex asymmetry of potential siRNA like products (Figure 1D). The lowest averaged level of remaining target RNA is achieved in the similar but narrower ΔG range from −32 to −28 kcal/mol. Results of shRNA experiments pre-designed for this study to have terminal duplex asymmetry of potential siRNA cleavage products above or equal to 2 kcal/mol are shown in Figure 1E. A similar categorization effect was observed for the new shRNA dataset; most efficient shRNAs, frequently were found exactly in the same ΔG value range from −32 to −28 kcal/mol. Detailed information for these siRNA and shRNAs datasets is presented in Table S1. To make sure that free energy values above −28 kcal/mol are not optimal we analyzed wider range of ΔG values and presented the results in Figure S2.

In our study, where shRNAs with optimal terminal duplex asymmetry and antisense strand target stability were included, two thirds of the molecules could reduce the mRNA level to 20% of the control level. Without this optimization, about one third of shRNAs could silence genes with the same efficiency [29]. Thus, our approach allows a significant improvement of efficient shRNAs design.

### Comparison of predictive power of duplex stability and other thermodynamic parameters

**Figure 2:**
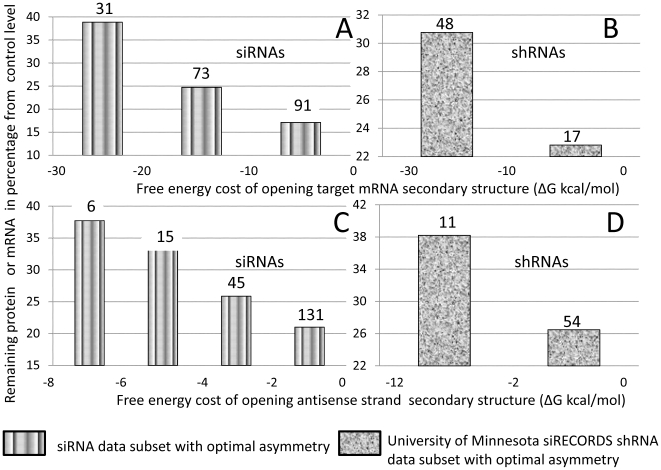
Relationships between silencing efficiency and stabilities of antisense strand and target secondary structures. Data points for shRNAs and siRNAs with optimal terminal duplex asymmetry (ΔΔG ≥2 kcal/mol) were combined from different experimental databases and further categorized according to stability of the target RNA secondary structure or the antisense strand secondary structure. The averaged level of the remaining protein or mRNA in cells was calculated for each category. Numbers of representatives in each data category are indicated above each column. The relevant correlation coefficient and significance values are 0.35 and 4.3*10^−7^ for relationships shown in the chart a, 0.1 and 0.4 for b, 0.23 and 8.9*10^−3^ for c, 0.32 and 0.008 for d. a. siRNAs categorized according to stabilities of target RNA secondary structures. b. shRNAs categorized according to stabilities of target RNA secondary structures. c. siRNAs categorized according to stabilities of the antisense strand secondary structures. d. shRNAs categorized according to stabilities of the antisense strand secondary structures.

Inspired by previously published studies [29], [30], [31], which demonstrated that secondary structure stability of target RNA can be used as a predictor of silencing efficiency, we performed data categorization according to this factor. Considering specificity and sensitivity of categorization we found that lowest average amount of remaining target RNA is achieved in the ΔG range from −10 to 0 kcal/mol. The results of this categorization for siRNAs and shRNAs and correlation coefficients between secondary structure stability of target RNAs and silence efficiency are presented in Figure 2A–B. Correlation coefficients, categorization data and results of ROC analysis presented in Table S2 did not demonstrate an advantage of using target RNA secondary structure stability instead of duplex stability of fully paired antisense strand of siRNA or shRNA molecules for prediction of silencing efficiency.

Data for siRNA and shRNA subsets with optimal terminal duplex asymmetry were also categorized according to the antisense strand secondary structure stability [25]. We found that lowest average amount of remaining target RNA is achieved in the ΔG range from −2 to 0 kcal/mol. The results of this categorization, correlation coefficients between this parameter and silencing efficiency (Figure 2C–D) and results of ROC analysis (Table S2) also did not demonstrate any significant advantage of using this factor instead of duplex stability of fully paired antisense strand of siRNA or shRNA molecules.

Regression analysis showed a strong correlation between duplex stability of fully paired siRNA antisense strand and stability of secondary structures of target RNA or stability of the antisense strand (Table S3). These data are in good agreement with published earlier results [3], [25]. This correlation explains our finding that combinations of all these parameters provided small categorization benefits (Table S4). It was shown earlier that variability of RNA secondary structure predictions could be minimized by using local RNA folding [30] with short window length (60 nt) for estimation of target site accessibility in the design of effective siRNAs. Local, rather than global RNA folding prediction better correlates with free energy of duplex stability and could be replaced by duplex stability in some cases [3], [32]. This is suggestive that duplex stability might reflect the level of target site accessibility with high accuracy. Taking into account high degree of variability in predictions of global RNA secondary structures and their high computational cost, we created software for efficient design of siRNAs and shRNAs based on the selection of candidates according to optimal duplex asymmetry and stability.

### Software for efficient siRNA and shRNA design which employ application of optimal thresholds for duplex asymmetry and stability

We developed a program “si-shRNA selector” that performs selection of efficient siRNA candidates. We use the term “siRNA” in the section describing the program regardless of whether the molecules originate from synthetic oligonucleotides transfected into cells or derived from enzymatic processing of shRNA. “si-shRNA selector” generates siRNA candidate oligonucleotides of user defined length. In the list of relevant duplexes generated from these oligonucleotides, it substitutes “C” to “U” for two 3′-terminal nucleotides of the sense strand. These substitutions generate G-U wobble pairing instead of more stable G-C pairing in the RNA duplex of the 5′ end of antisense strand. “si-shRNA selector” calculates terminal asymmetry of siRNA and siRNA-like duplex stability and removes candidates with strand asymmetry below 2 kcal/mol. From the remaining list it removes candidates with non-optimal siRNA duplex stability. The optimal range of duplex stability can be user defined. The optimal ranges suggested in this study are −35 to −27 kcal/mol for siRNAs transfected as oligonucleotides and −33 to −28 kcal/mol for siRNA-like duplexes produced from shRNA hairpins.

The program also removes sequences with “AAAA”, “TTTT”, “GGGG”, “CCCC”, “TGGC” motifs from siRNA efficient candidates. It is known that a stretch of four or more Ts is a termination signal for polymerase 3 which generates shRNAs, so such motifs need to be avoided in shRNA design. Also to be avoided are stretches of “A” complementary to runs of “T” in hairpin stems. We believe that homogeneous stretches of “A” or “T” are not beneficial for chemically synthesized siRNAs as well. Moreover, we suspect that runs of 4 or more of “G” or “C” are not beneficial for both siRNAs and shRNAs, although experimental tests are needed to verify or refute this point. The motif “TGGC” is removed because a strong correlation between its presence in siRNA and reduced cell viability, has been found [33]. The program output selects siRNAs as oligonucleotides for the “sense” and “antisense” strands without overhangs. The program is available at ftp://ftp.ncbi.nlm.nih.gov/pub/shabalin/siRNA/si_shRNA_selector/.

To date, relative efficiency of siRNA predicting software has been compared using correlation analysis and ROC curves [10]. Comparison revealed that siRNA scales, Biopredsi and DSIR software are similar in respect to high discrimination ability of efficient siRNAs [10]. More precisely, ROC curves corresponding to these software variants display similar prediction specificity/sensitivity tradeoffs [10]. Taking into account this similarity, we presented only the results of comparison of si-shRNA selector with siRNA scales in this study (Figure S3). Under default parameters settings, which correspond to high specificity/low sensitivity prediction tradeoff, our method performs similar to siRNA scales for siRNA design. However, the new method is better for shRNA predictions. For genome-wide analysis, when prediction of small numbers of highly efficient sh/siRNAs for each mRNAs in genome is desirable, this tradeoff is also efficient. Thus, using just two thermodynamic parameters, we developed a computer program that performs efficiently for both shRNA and siRNA predictions at the genomic level; producing results that are comparable with those of advanced software for siRNA design. The advantages of our approach are in simple calculations and clear physical understanding of thermodynamic parameters that allow designing candidates with predicted high strand selectivity for RISC loading and with high target site accessibility.

## Discussion

“si-shRNA selector” is optimal for mammalian shRNA design mainly since the database from which algorithm was designed includes experiments performed in mammalian cell cultures.

For efficient gene silencing, RNA hairpin transcripts need to be processed into siRNA or miRNA-like cleavage products that can enter RISC. The processing patterns of hairpin structured transcripts derived from relevant vectors could be different. It should be taken into account for appropriate application of “si-shRNA selector”.

Transcription of shRNAs is usually performed from Polymerase III promoters H1 or U6 [12], [34]. In this study, we used the H1 promoter which favors adenine at the first position of potential encoded shRNA or stem-loop construct. Under the H1 promoter, the cleavage guidance (antisense) strand could be positioned at the 5′ or 3′ halves of the stem in the relevant constructs without any sequence restrictions.

In case of U6 promoter is used it should be taken into consideration that it has a preference for guanine at the first position of the transcript. Positioning the antisense strand at the 3′ half of the hairpin is preferable under the U6 promoter with guanine at the first position, in order to achieve optimal duplex asymmetry. Alternatively, when the antisense strand needs to be positioned in the 5′ half of the stem, the “U” has to be positioned as the stem's last nucleotide to generate a “G-U” pairing. “si-shRNA selector” makes such wobble pairs automatically.

**Figure 3:**
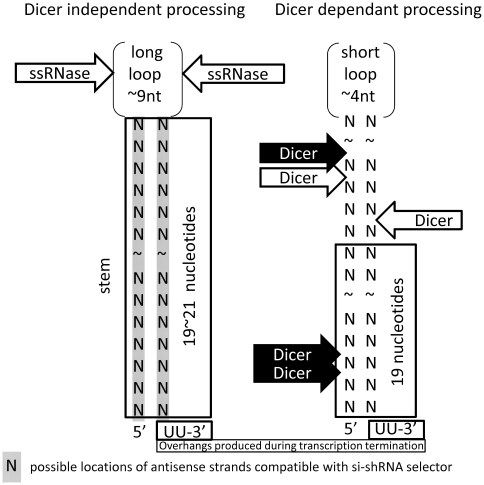
Variants of enzymatic processing of hairpin transcripts. Arrows with black background and white font show Dicer cleavage locations derived from one study [37], while arrows with white background and black font represent results from another [19]. “SsRNAse” is an abbreviation for single strand nucleases.

Hairpin structures with short 19–20 nucleotides stems and 9 nucleotides “UUCAAGAGA” loops were used in this study for shRNA design, which was described as the optimal configuration for a potent silencing trigger [35]. Such short stems might be not preferred substrates for Dicer, and these structures are likely processed by single strand specific RNases as suggested in earlier study [19]. These RNases are usually able to efficiently cleave stretches of pyrimidines, particularly uridines [20], [36]. It was experimentally shown that constructs with pyrimidine rich loop sequence “CTTCCTGTCA” and short hairpin stems (19 nucleotides) are also highly efficient and could be used for successful shRNA design (P.M. Chumakov personal communication). The hairpin structures with short stems (18–21 nt) and non-paired pyrimidines and particular uridines in the loops are optimal for the design of efficient molecules using siRNA-shRNA selector since they can form predictable duplexes after RNase processing (Figure 3).

shRNA constructs with micro RNA (miRNA) elements are becoming increasingly popular for loss-of-function genetic screens [17], [18]. However, the possibility that Dicer can cleave these hairpin structures in alternative ways is largely ignored. It is frequently assumed that Dicer cleaves ∼22 nucleotides from the base of the stem in hairpins with 2–3 U residues comprising 3′ overhangs. This type of Dicer cleavage certainly can occur [19], however, some nucleotides from the 5′ ends can be cleaved with equal efficiency [37]. Constructs with miRNA elements sometimes require Drosha for processing. Drosha cleavage specificity is also difficult to predict with great certainty. Moreover, variable 3′ overhangs can be formed as a result of Drosha cleavage and this variation can further influence Dicer specificity [37]. Consequently, hairpin structures that are Dicer and Drosha substrates are less optimal for the design of efficient candidates by “siRNA-shRNA selector” as they form variable and frequently unpredictable duplexes after enzymatic cleavage. Alternative processing of hairpin transcripts by different RNA nucleases is shown in Figure 3.

Optimization of the terminal asymmetry in siRNA duplexes increases both efficiency and specificity of silencing because it preferentially enhances entry of the guidance strand instead of the passenger strand, into RISC. Entry of the passenger strand into RISC can contribute to non specific cleavages. Terminal duplex wobble pairs or nucleotide chemical modifications can be designed to increase the number of molecules with optimal terminal duplex asymmetry. However, these approaches are not readily applicable to shRNA design. Substitutions for optimization of duplex asymmetry can change shRNAs enzymatic cleavage patterns, while nucleotide chemical modifications can't be performed *in vivo.*

The duplex stability threshold for the fully paired antisense strands of efficient siRNAs was discussed recently [38]. It was found that exclusion of siRNAs that form stable duplexes (ΔG less than −34.6 kcal/mol) improves the accuracy of different siRNA prediction models [38]. Thermodynamic evaluation of duplex stability using nearest neighbor parameters is more accurate than those based on evaluation of GC content. We found that siRNA duplexes with identical GC content have different ΔG values with variations as high as 4 kcal/mol.

Why use thermodynamic parameter thresholds instead of weights in predictive models? The disadvantage of regression analysis and similar approaches for predicting molecular efficiency are weight values assigned to each input variable to generate the predictor of siRNA efficiency in the output. These weight values can change as a reflection of concentration changes of siRNA or mRNA components in the cleavage reaction. As a result, weights that are found to be optimal based on the analysis of one experimental database might be far from optimal for another database. Results of regression analysis or similar approaches could be difficult to extrapolate on shRNA design, especially for viral transduction of shRNA constructs. In this case, the cellular concentration of the expected siRNA–like cleavage products is most likely smaller than with plasmid or oligonucleotide transfections. Threshold parameters might be adjusted to be less dependent on concentration changes and can be more universal in predicting molecular behavior under different experimental settings.

Why does duplex stability of the fully paired siRNA antisense strand affect silencing efficiency? This parameter should not be too high or too low for efficient siRNA functioning. Low duplex stability results in slow formation and short life time of antisense strand-target duplexes, which could be insufficient for RNA cleavage to occur. On the other hand, accessibility of GC rich siRNA antisense strand and complementary target mRNA for hybridization is diminished due to the high probability of self-interactions and stable local secondary structures. It is in good agreement with our results demonstrating that stability of siRNA duplex with fully paired strands strongly correlates with two other parameters, such as siRNA antisense and target mRNA secondary structure stabilities (Table S3). Perhaps this correlation is responsible for low average silencing activity of molecules with high duplex stability of the fully paired antisense strand.

Conclusion: We demonstrate that shRNA and siRNA molecules selected for optimal duplex terminal asymmetry and optimal duplex stability of fully paired antisense strand are, on average, highly efficient. We suggest a simple method assisting for the design of efficient shRNAs and siRNAs as well as software that implements this method.

## Supporting Information

Figure S1 Relationship between ratios of reactions rates and terminal duplex asymmetry. Thermodynamic model for interaction between siRNA duplex and RISC.
(0.20 MB DOC)

Figure S2 Relationship between silencing activity and duplex stability.
(0.07 MB DOC)

Figure S3 ROC analyses for individual and combined siRNA and shRNA databases.
(0.73 MB DOC)

Table S1 Supporting information to Figure1 and Figure 2.
(0.12 MB DOC)

Table S2 Statistical characteristics for relationships between si-shRNA silencing efficiency and RNA stabilities.
(0.05 MB DOC)

Table S3 Relationships between thermodynamic parameters.
(0.03 MB DOC)

Table S4 Effects of combination of optimized parameters calculated from siRNA and shRNA experimental databases.
(0.04 MB DOC)
