# Supplementary material for: Optimization of Duplex Stability and Terminal Asymmetry for shRNA Design
Source: PLoS One. 2010 Apr 20;5(4):e10180. doi: 10.1371/journal.pone.0010180 (PMC2857877; doi:10.1371/journal.pone.0010180)
Supplement: Figure S1 — Relationship between ratios of reactions rates and terminal duplex asymmetry. Thermodynamic model for interaction between siRNA duplex and RISC. (0.20 MB DOC) [file pone.0010180.s001.doc]

**Supplementary materials**

**Figure S1. Relationship between ratios of reactions rates and terminal duplex asymmetry.**

Experimentally determined ratios (target cleavage directed with one strand versus another) are shown as white diamonds. Experimental ratios were calculated from Figure 2 (panels B, H, J, K and L in Schwarz, et al., 2003 [2]. Calculations were done at seven minutes time point after reaction start. Estimations of siRNA/RISC reaction rate ratios (RISC loading with one siRNA strand versus another) based on Eyring’s transition state theory (<http://www.owlnet.rice.edu/ ~chem445/lec09.pdf>, paragraph 2.3, page 2) are shown as black squares.

**Thermodynamic model for interaction between siRNA duplex and RISC**

Here we consider intermediate stages in siRNA-directed cleavage reactions to understand why the reaction rates are so variable for different siRNAs and what is the relationship between the rates of siRNA directed cleavage reaction and the terminal stabilities of siRNA duplexes. We propose a model which applies the “transition state theory” (<http://www.owlnet.rice.edu/~chem445/lec09.pdf>) to the process^1^. The first stage of siRNA directed cleavage is the reversible absorption of the antisense strand into the RISC, i.e. "transition" complex formation. This stage is followed by further nonreversible reactions resulting in products such as cleaved siRNA duplex sense strand and cleaved target mRNA. These have the following form:

*K_a_* *k*

[SA]+E 🡨🡪[AES] 🡪[AE]+S^*^ (1)

Overall reaction

*k_r_*

[AE]+mRNA 🡪 [AE]+mRNA^*^ (2)

*k_a_*

[SA]+E+mRNA 🡪[AE]+mRNA^*^+S^*^ (3)

Here [SA] denotes the duplex of the sense and antisense strands (siRNA duplex), E – the enzyme, [AES] – the transition complex, [AE] – the RISC loaded with the antisense strand, S^*^ and mRNA^*^ – the cleaved sense strand and the target mRNA (reaction products); *k_s_* *k_r_*, *k_a_* – the kinetic constants, corresponding to reactions (1), (2) and (3).

Our model predicts a linear relationship of the logarithm of the experimental reaction rate and the energy related to stability the terminal nucleotide base pairs. The two strands of the siRNA duplex can participate in RISC formation and compete with each other. So two parallel reactions can occur and lead to the formation of two different product types. One product type is RISC loaded with antisense strand (see (3)) and the other is RISC loaded with siRNA duplex sense strand (4). For this reaction we can write a similar equation:

*k_s_*

[SA]+E+mRNA 🡪 [SE]+mRNA^*^+A^*^ (4)

Here [SE] is the RISC loaded with the antisense strand, A^*^ and mRNA^*^ are the cleaved sense strand and non-target mRNA (reaction products); *k_s_* – the kinetic constant, corresponding to this reaction.

We assume that the formation of the transition complex is a rate limiting stage for reaction (3). The formation of transition complex [AE S] is characterized by the equilibrium constant of binding K_a_. The logarithm of this constant is related to the free energy of transition complex formation. The first stage of the formation of this complex is the melting of the siRNA terminal duplex. The efficiency of this stage is determined by the terminal stabilities of the siRNA duplex. These stabilities, in turn, are determined by the free energy of terminal nucleotides in the duplex. It is likely that the energy related to the stability of terminal 5’ nucleotides pairs in an siRNA duplex is part of the energy of formation of the RISC transition state. Accordingly, the energy of formation of the transition complex depends linearly on the energy related to stability of terminal 5’ nucleotide base pairs in a siRNA duplex. Estimation of the energy required for transition complex formation can be performed using experimental data^2^. According to the assumption that the formation of RISC is a rate limiting stage for reaction (3), the logarithm *k*_a_ will depend linearly on the energy of transition complex formation (ΔF_a_):

ln *k*_a_ = - ΔF_a_ /RT + const (5)

The logarithm of the reaction rate (3) depends on the free energy of formation of the transition complex, and this energy includes the energy related to stability of the 5’ antisense strand terminus of siRNA:

ΔF_a_ /RT = C_1_ ΔG_a_ /RT + C_2_ (6)

Here ΔG_a_ is the energy related to the stability of the 5’ antisense strand terminus of siRNA; C_1_ and C_2_ are constants. Putting (5) and (6) together, we get:

ln k_a_ = - C_1_ ΔG_a_ /RT - C_2_ + const (7)

Equation (8) can be derived in a same way as (7):

ln *k*_s_ = - C_1_ ΔG_s_ /RT + const (8)

From (7) and (8) the next equation (9) can be derived:

ln *k*_a_ / *k*_s_ = - C_1_ ΔΔG /RT + const (9)

Here ΔΔG = ΔG_a_ - ΔG_s_

ΔΔG is the difference between the ∆G stability of the 5’ ends of the antisense and sense strands. Thus this model predicts a linear relationship between ΔΔG and log-ratio of the rates of the two competing reactions.

References

1. Neil Issacs, Physical Organic Chemistry, Second Edition, by Addison Wesley Longman, (1995).

2. Schwarz, D.S. *et al*. Asymmetry in the assembly of the RNAi enzyme complex. *Cell* **115**, 199-208 (2003).
