# Supplementary material for: Optimization of Duplex Stability and Terminal Asymmetry for shRNA Design
Source: PLoS One. 2010 Apr 20;5(4):e10180. doi: 10.1371/journal.pone.0010180 (PMC2857877; doi:10.1371/journal.pone.0010180)
Supplement: Figure S2 — Relationship between silencing activity and duplex stability. (0.07 MB DOC) [file pone.0010180.s002.doc]

**Figure S2. Relationship between silencing activity and duplex stability.** Silencing experiments were categorized according to RNA duplex stability measured as ΔG. The average amount of target mRNA or remaining relevant protein was calculated for each category. All data points with ΔΔG above 1 kcal/mol from four different siRNA databases (siRNAs) and shRNA experiments (shRNAs; from siRECORDS University of Minnesota database) were analyzed in this study.

**
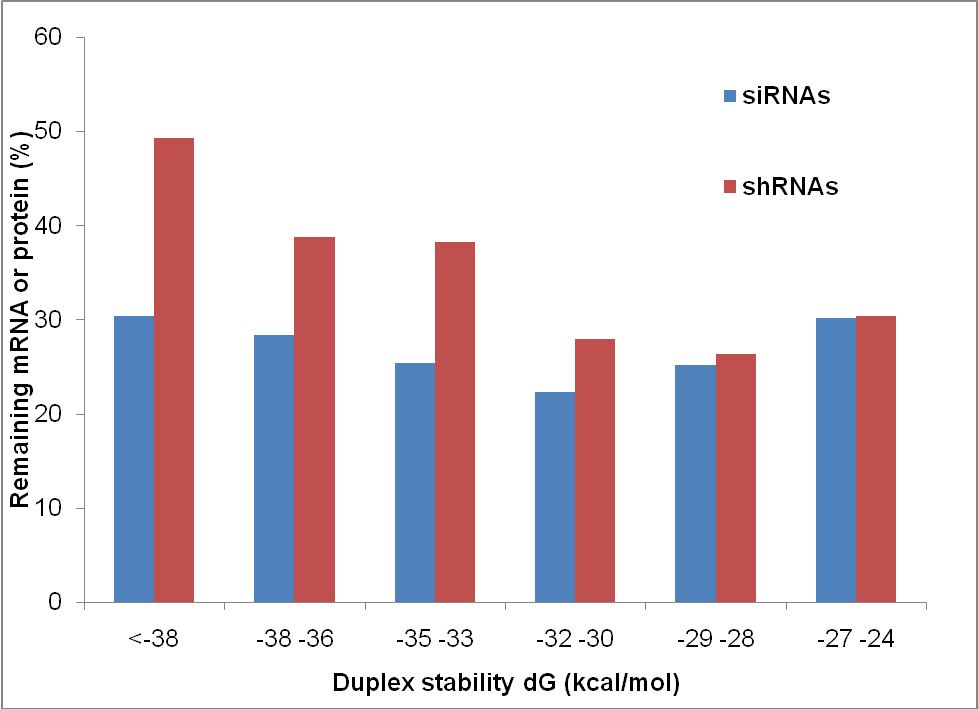
**
