# Supplementary material for: Optimization of Duplex Stability and Terminal Asymmetry for shRNA Design
Source: PLoS One. 2010 Apr 20;5(4):e10180. doi: 10.1371/journal.pone.0010180 (PMC2857877; doi:10.1371/journal.pone.0010180)
Supplement: Figure S3 — ROC analyses for individual and combined siRNA and shRNA databases. (0.73 MB DOC) [file pone.0010180.s003.doc]

**Figure S3. ROC analyses for individual and combined siRNA and shRNA databases.** Scoring scheme for ROC analysis allows two possibilities for representing si-shRNA selector filters:

- - Filter 1: ddG >= 2 kcal/mol and dG outside optimal range
  - Filter 2: ddG >=2 kcal/mol and dG in optimal ranges
    - -35 kcal/mol <= dG <= -27 kcal/mol for siRNAs
    - -32 kcal/mol <= dG <= -28 kcal/mol for shRNAs

**All siRNA databases combined**

**All shRNA databases combined**

**Novartis siRNAs**

**Sloan Kettering siRNAs**

**University of Tokio siRNAs**

**NCBI siRNAs**

**University of Minnesota shRNAs**

**Princeton University shRNAs**

**Netherlands Cancer Center shRNAs**
