# Supplementary material for: Optimization of Duplex Stability and Terminal Asymmetry for shRNA Design
Source: PLoS One. 2010 Apr 20;5(4):e10180. doi: 10.1371/journal.pone.0010180 (PMC2857877; doi:10.1371/journal.pone.0010180)
Supplement: Table S1 — Supporting information to Figure1 and Figure 2. (0.12 MB DOC) [file pone.0010180.s004.doc]

**Table S1.** Supporting information to Figure1 and Figure 2.

| Figure  1a | X Interval (∆∆G in kcal/mol) from | Novartis database | -3 | -2 | -1 | 0 | 1 | 2 |
| --- | --- | --- | --- | --- | --- | --- | --- | --- |
|  | to |  | -2 | -1 | 0 | 1 | 2 | 3 |
|  | Number of the data-points in the X interval |  | 97 | 493 | 703 | 486 | 551 | 101 |
|  | Y (average in the interval) |  | 54.15 | 42.62 | 32.26 | 27.15 | 21.27 | 19.99 |
|  | Y (min in the interval) |  | 21.3 | 0.1 | 0.1 | 0.1 | 0.1 | 0.1 |
|  | Y (max in the interval) |  | 99.8 | 89.5 | 100 | 91.4 | 78.2 | 63.9 |
|  |  |  |  |  |  |  |  |  |
|  | Number of the data-points in the X interval | Sloan Kettering database | 29 | 128 | 157 | 112 | 139 | 36 |
|  | Y (average in the interval) |  | 80.83 | 64.55 | 56.05 | 42.87 | 39.7 | 27.26 |
|  | Y (min in the interval) |  | 6 | 4 | 4 | 0.1 | 1 | 0.1 |
|  | Y (max in the interval) |  | 100 | 100 | 100 | 100 | 94 | 89 |
|  |  |  |  |  |  |  |  |  |
|  | Number of the data-points in the X interval | University of Tokyo database | 25 | 142 | 208 | 148 | 154 | 25 |
|  | Y (average in the interval) |  | 61.56 | 54.25 | 46.44 | 44.6 | 40.01 | 32.14 |
|  | Y (min in the interval) |  | 6.1 | 8.3 | 6.3 | 2 | 4 | 3.3 |
|  | Y (max in the interval) |  | 94 | 96.2 | 100 | 96.2 | 94.9 | 74.9 |
|  |  |  |  |  |  |  |  |  |
|  | Number of the data-points in the X interval | NCBI database | 17 | 117 | 215 | 131 | 132 | 41 |
|  | Y (average in the interval) |  | 72.76 | 60.27 | 44.72 | 38.29 | 29.21 | 23.45 |
|  | Y (min in the interval) |  | 21.4 | 1 | 2 | 0 | 1 | 2.67 |
|  | Y (max in the interval) |  | 118.58 | 127 | 120 | 127.8 | 110 | 67 |
|  |  |  |  |  |  |  |  |  |
| Figure 1b | X Interval (∆∆G in kcal/mol) from | siRecords database | -3 | -2 | -1 | 0 | 1 | 2 |
|  | to |  | -2 | -1 | 0 | 1 | 2 | 3 |
|  | Number of the data-points in the X interval |  | 13 | 78 | 218 | 142 | 227 | 68 |
|  | Y (average in the interval) |  | 50.77 | 42.31 | 36.74 | 36.48 | 33.33 | 29.68 |
|  | Y (min in the interval) |  | 5 | 5 | 5 | 5 | 5 | 5 |
|  | Y (max in the interval) |  | 100 | 100 | 100 | 100 | 100 | 100 |

|  |  |  |  | |  | |  | |  | |  | |  | |  |
| --- | --- | --- | --- | --- | --- | --- | --- | --- | --- | --- | --- | --- | --- | --- | --- |
| Figure 1c | X Interval (∆G in kcal/mol) from | siRNA subset with ∆∆G≥2 kcal/mol | -55 | | -40 | | -35 | |  | |  | |  | |  |
|  | to |  | -40 | | -35 | | -27 | |  | |  | |  | |  |
|  | Number of the data-points in the X interval |  | 68 | | 72 | | 57 | |  | |  | |  | |  |
|  | Y (average in the interval) |  | 26.21 | | 23.98 | | 21.8 | |  | |  | |  | |  |
|  | Y (min in the interval) |  | 0.1 | | 0.1 | | 0.1 | |  | |  | |  | |  |
|  | Y (max in the interval) |  | 71.5 | | 89 | | 86 | |  | |  | |  | |  |
|  |  |  |  | |  | |  | |  | |  | |  | |  |
| Figure 1d | X Interval (∆G in kcal/mol) from | siRecords subset with ∆∆G≥2 kcal/mol | -40 | | -35 | | -32 | |  | |  | |  | |  |
|  | to |  | -36 | | -32 | | -28 | |  | |  | |  | |  |
|  | Number of the data-points in the X interval |  | 8 | | 25 | | 32 | |  | |  | |  | |  |
|  | Y (average in the interval) |  | 35.62 | | 30.2 | | 25 | |  | |  | |  | |  |
|  | Y (min in the interval) |  | 5 | | 5 | | 5 | |  | |  | |  | |  |
|  | Y (max in the interval) |  | 100 | | 100 | | 100 | |  | |  | |  | |  |
|  |  |  |  | |  | |  | |  | |  | |  | |  |
| Figure 1e | X Interval (∆G in kcal/mol) from | Princeton database | -40 | | -36 | | -32 | |  | |  | |  | |  |
|  | to |  | -36 | | -32 | | -28 | |  | |  | |  | |  |
|  | Number of the data-points in the X interval |  | 31 | | 45 | | 7 | |  | |  | |  | |  |
|  | Y (average in the interval) |  | 65.88 | | 53.18 | | 25.71 | |  | |  | |  | |  |
|  | Y (min in the interval) |  | 4 | | 0 | | 0 | |  | |  | |  | |  |
|  | Y (max in the interval) |  | 100 | | 100 | | 50 | |  | |  | |  | |  |
|  |  |  | |  | |  | |  | |  | |  | |  | |
| Figure 2a | X Interval (∆G in kcal/mol) from | siRNA subset with ∆∆G≥2 kcal/mol | | -30 | | -20 | | -10 | |  | |  | |  | |
|  | to |  | | -20 | | -10 | | 0 | |  | |  | |  | |
|  | Number of the data-points in the X interval |  | | 31 | | 73 | | 91 | |  | |  | |  | |
|  | Y (average in the interval) |  | | 38.88 | | 24.75 | | 17.14 | |  | |  | |  | |
|  | Y (min in the interval) |  | | 3 | | 0.1 | | 0.1 | |  | |  | |  | |
|  | Y (max in the interval) |  | | 89 | | 74.9 | | 53.5 | |  | |  | |  | |
|  |  |  | |  | |  | |  | |  | |  | |  | |
| Figure 2b | X Interval (∆G in kcal/mol) from | siRecords subset with ∆∆G≥2 kcal/mol | | -30 | | -10 | |  | |  | |  | |  | |
|  | to |  | | -10 | | 0 | |  | |  | |  | |  | |
|  | Number of the data-points in the X interval |  | | 48 | | 17 | |  | |  | |  | |  | |
|  | Y (average in the interval) |  | | 31.48 | | 22.65 | |  | |  | |  | |  | |
|  | Y (min in the interval) |  | | 5 | | 5 | |  | |  | |  | |  | |
|  | Y (max in the interval) |  | | 100 | | 100 | |  | |  | |  | |  | |
|  |  |  | |  | |  | |  | |  | |  | |  | |
| Figure 2c | X Interval (∆G in kcal/mol) from | siRNA subset with ∆∆G≥2 kcal/mol | | -8 | | -6 | | -4 | | -2 | |  | |  | |
|  | to |  | | -6 | | -4 | | -2 | | 0 | |  | |  | |
|  | Number of the data-points in the X interval |  | | 6 | | 15 | | 45 | | 131 | |  | |  | |
|  | Y (average in the interval) |  | | 37.75 | | 31.71 | | 26.09 | | 21.01 | |  | |  | |
|  | Y (min in the interval) |  | | 22.8 | | 6.7 | | 0.1 | | 0.1 | |  | |  | |
|  | Y (max in the interval) |  | | 53.5 | | 74.9 | | 86 | | 89 | |  | |  | |
|  |  |  | |  | |  | |  | |  | |  | |  | |
| Figure 2d | X Interval (∆G in kcal/mol) from | siRecords subset with ∆∆G≥2 kcal/mol | | -12 | | -2 | |  | |  | |  | |  | |
|  | to |  | | -2 | | 0 | |  | |  | |  | |  | |
|  | Number of the data-points in the X interval |  | | 11 | | 54 | |  | |  | |  | |  | |
|  | Y (average in the interval) |  | | 38 | | 26.76 | |  | |  | |  | |  | |
|  | Y (min in the interval) |  | | 5 | | 5 | |  | |  | |  | |  | |
|  | Y (max in the interval) |  | | 100 | | 100 | |  | |  | |  | |  | |
