# Supplementary material for: Optimization of Duplex Stability and Terminal Asymmetry for shRNA Design
Source: PLoS One. 2010 Apr 20;5(4):e10180. doi: 10.1371/journal.pone.0010180 (PMC2857877; doi:10.1371/journal.pone.0010180)
Supplement: Table S2 — Statistical characteristics for relationships between si-shRNA silencing efficiency and RNA stabilities. (0.05 MB DOC) [file pone.0010180.s005.doc]

**Table S2. Statistical characteristics for relationships between si-shRNA silencing efficiency and RNA stabilities.** Significant values (P<0.05) are highlighted in yellow.

| data subset with optimal terminal duplex asymmetry  (∆∆G≥2 kcal/mol) | duplex | RNA secondary structure | antisense strand secondary structure |
| --- | --- | --- | --- |
| **siRECORDS UM** (correlation coefficient (R)) | -0.26719 | -0.09735 | -0.31768 |
| Correlation significance level (P) | 0.028828 | 0.433189 | 0.008803 |
| Area under the ROC curve (AUC) | 0.61 | 0.572 | 0.532 |
| Standard error | 0.0722 | 0.0742 | 0.0758 |
| 95% Confidence interval | 0.483 to 0.727 | 0.446 to 0.693 | 0.406 to 0.655 |
| z statistic | 1.526 | 0.976 | 0.423 |
| Significance level P (Area=0.5) | 0.127 | 0.3291 | 0.672 |
| **Novartis (**correlation coefficient) | -0.35581 | -0.27312 | -0.38292 |
| Correlation significance level P | 0.000261 | 0.005719 | 7.74E-05 |
| Area under the ROC curve (AUC) | 0.661 | 0.625 | 0.661 |
| Standard error | 0.0537 | 0.0553 | 0.0537 |
| 95% Confidence interval | 0.560 to 0.752 | 0.524 to 0.720 | 0.560 to 0.753 |
| z statistic | 2.998 | 2.269 | 3.003 |
| Significance level P (Area=0.5) | 0.0027 | 0.0233 | 0.0027 |
| **Sloan Kettering** (correlation coefficient) | -0.30199 | -0.37668 | 0.020324 |
| Correlation significance level P | 0.066231 | 0.036731 | 0.760749 |
| Area under the ROC curve (AUC) | 0.696 | 0.642 | 0.537 |
| Standard error | 0.0959 | 0.101 | 0.105 |
| 95% Confidence interval | 0.505 to 0.847 | 0.450 to 0.805 | 0.350 to 0.717 |
| z statistic | 2.043 | 1.409 | 0.357 |
| Significance level P (Area=0.5) | 0.0411 | 0.1589 | 0.7213 |
| **Tokyo University** (correlation coefficient) | -0.62494 | -0.2317 | -0.57275 |
| Correlation significance | 0.000838 | 0.265085 | 0.002769 |
| Area under the ROC curve (AUC) | 0.844 | 0.733 | 0.792 |
| Standard error | 0.0905 | 0.111 | 0.102 |
| 95% Confidence interval | 0.643 to 0.956 | 0.519 to 0.888 | 0.583 to 0.926 |
| z statistic | 3.798 | 2.099 | 2.872 |
| Significance level P (Area=0.5) | 0.0001 | 0.0358 | 0.0041 |
| **NCBI** (correlation coefficient) | -0.28064 | -0.2229 | -0.09995 |
| Correlation significance level P | 0.075512 | 0.161261 | 0.534114 |
| Area under the ROC curve (AUC) | 0.573 | 0.53 | 0.636 |
| Standard error | 0.09 | 0.0912 | 0.0868 |
| 95% Confidence interval | 0.409 to 0.726 | 0.368 to 0.687 | 0.472 to 0.781 |
| z statistic | 0.811 | 0.328 | 1.572 |
| Significance level P (Area=0.5) | 0.4176 | 0.7429 | 0.1161 |
|  |  |  |  |
