# Supplementary material for: Optimization of Duplex Stability and Terminal Asymmetry for shRNA Design
Source: PLoS One. 2010 Apr 20;5(4):e10180. doi: 10.1371/journal.pone.0010180 (PMC2857877; doi:10.1371/journal.pone.0010180)
Supplement: Table S3 — Relationships between thermodynamic parameters. (0.03 MB DOC) [file pone.0010180.s006.doc]

**Table S3**. Relationships between thermodynamic parameters.

|  | Correlation coefficients for relationship between antisense strand – target duplex stability evaluated through ∆G calculation versus | |
| --- | --- | --- |
| Database | target secondary structure stability evaluated through ∆G calculation | antisense secondary structure stability evaluated through ∆G calculation |
| Novartis | 0.57 (p=5.4*10^-212^) | 0.54 (p=3.5*10^-183^) |
| Sloan Kettering | 0.27 (p=9.7*10^-11^) | 0.49 (p=5.0*10^-24^) |
| University of Tokyo | 0.49 (p=2.8*10^- 44^) | 0.51 (p=3.54*10^-47^) |
| NCBI | 0.51 (p=2.4*10^-44^) | 0.48 (p=1.4*10^-43^) |
