# Supplementary material for: Optimization of Duplex Stability and Terminal Asymmetry for shRNA Design
Source: PLoS One. 2010 Apr 20;5(4):e10180. doi: 10.1371/journal.pone.0010180 (PMC2857877; doi:10.1371/journal.pone.0010180)
Supplement: Table S4 — Effects of combination of optimized parameters calculated from siRNA and shRNA experimental databases. (0.04 MB DOC) [file pone.0010180.s007.doc]

**Table S4. Effects of combination of optimized parameters calculated from siRNA and shRNA experimental databases.**

|  | Name of the category | | | | | |
| --- | --- | --- | --- | --- | --- | --- |
|  | No selection | ∆∆G of terminal duplex asymmetry ≥ 2kcal/mol and more | | | | |
|  |  | No selection | Optimal ∆G antisense-target duplex stability kcal/mol | ∆G antisense strand secondary structure stability above -2 kcal/mol | ∆G of target secondary structure  stability  above -10 kcal/mol | All categories overlap |
| number of representatives in the category of experiments from Novartis, Sloan Kettering, University of Tokyo and NCBI combined databases | 4386 | 198 | 64 | 131 | 91 | 22 |
| average remaining protein level in the category | 37% | 25% | 20% | 21% | 18% | 16.5% |
| representatives in the category that diminishing protein level to 30% or less | 43% | 65% | 81% | 76% | 87% | 90% |
| representatives in the category that diminishing protein level to 10% or less | 13% | 28% | 41% | 34% | 37% | 50% |
| how many target sites from 1000, fits the category | 1000 | 45 | 14 | 29 | 21 | 5 |
|  |  |  |  |  |  |  |
| number of representatives in the category of experiments from, University of Minnesota siRecords shRNA database | 733 | 68 | 32 | 54 | 17 | 9 |
| representatives in the category that diminishing protein level to 20% or less | 31% | 67% | 75% | 71% | 84% | 78% |
